# Modern Methods of Physical Treatment in Lung Diseases

**Published:** 1913-05-31

**Authors:** 


### NEW DEVELOPMENTS IN MEDICINE.

### V.—Modern Methods of Physical Treatment in Lung Diseases.

Modern physiology has during the last ten years almost recast its views with regard to the interesting and much-discussed question of respiration. While it would take up too much space to deal, even briefly, with this question, it may be stated that during recent years we have learned to appreciate the fact that the regulation of the function of respiration depends on the need of the tissues for oxygen, and not, primarily, on the amount of carbonic acid in the tissues themselves. We now know that carbonic acid is a necessity for maintaining the rhythm of respiration. This is shown by the remarkable experiment in which a curarised animal is rendered apnœic by section of the nerves of respiration. Such an animal rapidly succumbs if left alone; if, however, a small stream of oxygen, mingled with a small amount of carbonic-acid gas, is passed into the lungs, the animal continues alive, even though it makes no visible respiratory movement. Similarly, an animal which is being kept alive by artificial respiration becomes apnœic directly the artificial respiration is stopped, but it recommences its breathing if it is transfused with a watery solution of $CO_2$. The apnœa seen in cases of tracheotomy, and the apnœa in cases of anæsthetic poisoning, occasionally responsible for a death, are probably due to the same cause—a deficiency of stimulating $CO_2$. The experiments and clinical observations of Goldstein, Rubner, and Galeotti have shown the importance of warmth and psychical and emotional influences upon the respiratory rhythm, and have further proved the importance of $CO_2$ as a "hormone" or chemical stimulatory factor in producing respiratory rhythm. The results of these experiments and observations have been felt in medicine, in so far as they have importantly modified certain lines of treatment. A mouth-breather, as Hofbauer has shown, is a superficial breather; the apices of his lungs are never properly ventilated, while the air that he takes in is never properly warmed, and its quantity of moisture never properly regulated. The mere removal of obstructions to nasal breathing is not usually sufficient to eradicate what has become a habit; exercise and physical treatment are often necessary, especially in cases where the lung alveoli have already suffered, and a condition of bronchitis or emphysema has been produced. The breathing of artificially heated and dried air, as suggested by Schmidt, has been found singularly effective in cases of bronchitis and bronchial catarrh; the air is heated by a small electrothermopile to 150° F. It is still a question how far the physician can succeed in directly acting upon the lungs by inhalatory methods. It is certain, however, that inspired air penetrates much more deeply into the lungs through nasal respiration than through mouth-breathing. Recent experiments have shown that finely divided vegetable particles breathed in produce greater disturbance in the lung alveoli than harder mineral particles, probably owing to the fact that the former are less sterile. Suspended globules of fluid penetrate much deeper. This fact has been utilised in the modern treatment of bronchial asthma by inhalation of vapour containing adrenalin. The best method is that of Toevolgyi, who applies the spray directly to the mucosa of the bronchus by means of a bronchoscope of the Bruning type. In the same disease the influence of a high altitude has been early acknowledged. Modern hospital cases are treated by producing in the ward an atmosphere which more or less approaches that of a mountain climate. This is produced by a special oxygen apparatus, which enables the patient to inhale oxygen in a rarefied state and at a comparatively low pressure. A further development in the treatment of asthma has been Singalin's rhythmoscope, which is used in combination with a series of specially designed respiratory exercises, and Kühne's face-mask, which is already well known in connection with the physical treatment of pulmonary tuberculosis. The influence of certain drugs upon the bronchial nerves and mucosa is well known, and is regularly used, in combination with modern physical methods, in the treatment of asthma and emphysematous bronchitis. Thus, Nowotny's formula (2 per cent. cocaine in a 1 per cent. solution of adrenalin 1 : 10) has proved singularly efficacious when the solution is sprayed directly into the bronchi. Other physicians combine atropine with this formula, and it is a well-known fact that most so-called secret cures for asthma contain this alkaloid in solution.

The treatment of emphysema has hitherto been exceedingly unsatisfactory. Indeed, it has been held by many physicians that a symptomatic treatment alone could afford the patient any relief. Now, however, it has been found that in certain cases much may be done by physical exercises, while in certain advanced cases active surgical interference holds out a reasonable chance of cure, or, at least, of such amelioration that the patient virtually declares himself cured. Freund, for example, has shown that in certain cases an emphysematous condition of the lung is the result of a more or less immobile chest-wall. In such cases his explanation is that the chondro-sternum and the rib cartilages are hardened and partly ossified; the thorax is, therefore, fixed and scarcely moves upon inspiration or expiration, with the result that the ventilation of the lungs is considerably interfered with. Stagnation of the air in the alveoli leads to a deep-seated bronchitis, which in course of time develops into a bronchiectasis; the efforts of the patient to expel the mucus and half-liquid contents of the enlarged alveoli in turn lead to a greater strain being thrown upon the heart, so that ultimately a vicious circle is produced. Freund, therefore, proposed that the surgeon should intervene, and by severing the rib cartilages should make one part of the chest-wall mobile. The operation has been carried out with success in several cases, and, although there is still considerable difference of opinion as regards the indications for such surgical interference, it may now be held that Freund's operation has justified itself and has won a well-deserved place in the treatment of this particular disease. Dealing with the whole question of the treatment of emphysema on modern lines, De la Camp, of Freiburg, in a recent article in the *Zeitschrift für Arztliche Fortbildung* (which gives an excellent account of the recent advances in the physiology of respiration), declares himself strongly against mechanical interference by massage movements, and against treatment by compressed air. He favours a modified form of pneumotherapy, which consists in improving the inspiratory musculature, the regular use, under medical supervision, of Kühne's mask, and the improvement of the diaphragmatic movements by hydrotherapeutic treatment. The low-pressure cabinet of Brauer has also been found of use.

In cases of lung tuberculosis, physical methods of treatment have proved their efficacy in various ways. The great lever here has been the induction of a state of collapse in the lung or portion of lung affected by producing an artificial pneumothorax. The discussion with regard to the advantages of this method of treatment has, therefore, centred around the question of lung collapse in general, a question which the work of Sauerbruch and Forlanini has done much to elucidate. At Freiburg the double apparatus of Deneke is used, and, according to De la Camp, the results have been most encouraging. We published a short account of the method of inducing artificial pneumothorax in cases of pulmonary tuberculosis in a recent article in THE HOSPITAL. One of the indications for this modern method of treatment is bleeding from the lung; another is certain forms of unilateral bronchiectasis that resist treatment by other methods. More recently Müller and Saxl have shown that hæmorrhage from the lungs can be well controlled by injections of calcium chloride in gelatine solution. When, owing to adhesions between pleura and lung, it is impossible to produce a satisfactory pneumothorax, resection of the ribs is indicated, the best method being the parasternal and paravertebral operation of Sauerbruch. Much more recently it has been suggested that a method applicable in cases of pulmonary phthisis is to cause partial collapse of the affected lung by ligature of the bronchial arteries supplying that portion; the method has so far only been tried experimentally on animals, but the technique, with the advantages of a special cabinet such as described in a preceding article, offers no particular difficulties to the surgeon. Yet a third method to obtain partial collapse is that of Stuertz, who divided the phrenic nerve on one side, and thus paralysed the diaphragm unilaterally.

Physical exercise and respiratory gymnastics are now recognised as excellent aids in the treatment of incipient cases of lung tuberculosis. On this all modern authorities lay great stress, for obviously the above-mentioned methods of treatment are applicable only to far advanced cases. It is, therefore, of the greatest importance that medical men should make their patients realise the evils that result from bad respiration, evils that in the majority of cases can be prevented by a little attention and care and by suitable physical exercises designed to aid the musculature of the patient with defective nasal breathing.

